# Genetic risk of alcohol-related liver cirrhosis: Associations of *PNPLA3*, *TM6SF2*, and a two-variant polygenic risk score

**DOI:** 10.17305/bb.2025.13261

**Published:** 2025-12-12

**Authors:** Branka Nesic, Marina Jelovac, Teodora Karan Djurasevic, Dusica Vrinic Kalem, Petar Svorcan, Branka Zukic, Ivana Grubisa

**Affiliations:** 1 Department for Human Genetics, University Hospital Medical Center “Zvezdara”, Belgrade, Serbia; 2 Laboratory for Molecular Biomedicine, Institute of Molecular Genetics and Genetic Engineering, Universitiy of Belgrade, Belgrade, Serbia; 3 Clinical Department for Gastroenterology and Hepatology, University Hospital Medical Center “Zvezdara”, Belgrade, Serbia; 4 Faculty of Medicine, University of Belgrade, Belgrade, Serbia

**Keywords:** Alcohol-related liver cirrhosis, ALC, genetic variants, *PNPLA3*, *TM6SF2*, polygenic risk score, PRS

## Abstract

A minority of individuals who consume excessive alcohol develop cirrhosis. Variants in the patatin-like phospholipase domain-containing protein 3 gene (*PNPLA3*) and the transmembrane 6 superfamily member 2 gene (*TM6SF2*) have been previously identified as associated with alcohol-related cirrhosis (ALC). This study aimed to examine the variants of *PNPLA3* and *TM6SF2* and to develop and assess a polygenic risk score (PRS) for ALC. We enrolled 118 patients diagnosed with ALC and 131 control subjects, who were either abstainers or low-level alcohol consumers without evidence of liver disease. Genotyping of risk variants was performed using PCR-RFLP methodology. PRS, based on independent allelic effect size estimates from genotyped genetic loci, were computed and compared across groups. The development of ALC was significantly associated with CG and GG genotypes of *PNPLA3* (CG: OR: 1.82; 95% CI: 1.05–3.17; *P* ═ 0.033; GG: OR: 7.64; 95% CI: 3.06–19.07; *P* < 0.001) and the CT genotype of *TM6SF2* (OR: 2.43; 95% CI: 1.27–4.63; *P* ═ 0.007), controlling for age and sex. Patients with cirrhosis exhibited a significantly higher mean PRS compared to controls (0.32 vs 0.167, *P* ═ 1.8e-07). The odds ratios (ORs) and 95% confidence intervals for the group with the highest PRS score compared to the reference group were 6.707; 95% CI: 3.313–13.581, *P* < 0.001. In our ALC patient cohort, the *PNPLA3* rs738409 and *TM6SF2* rs58542926 variants were associated with an increased risk of ALC development. Moreover, the PRS derived from these two variants effectively identified the genetic components linked to cirrhosis within the study population.

## Introduction

Excessive alcohol consumption is a primary contributor to prolonged hepatic inflammation, which can progressively advance from alcohol-related fatty liver to alcoholic steatohepatitis, and ultimately to alcohol-related cirrhosis (ALC) in 10%–15% of long-term excessive drinkers [1]. Cirrhosis is an irreversible and progressive condition associated with high mortality rates due to liver failure and a significant incidence of hepatic malignancies [2]. This multifactorial disease is influenced by genetic and environmental risk factors and their interactions. In Europe, nearly 60% of cirrhosis cases are attributed to alcohol consumption [3].

Risk factors for the onset and progression of ALC include the quantity and pattern of alcohol intake, as well as the differential impact of alcohol on males and females [4–6]. Additionally, substantial interindividual variability in disease susceptibility is associated with genetic components [7, 8].

Recent investigations have focused on variations in several genes involved in alcohol metabolism, ethanol-induced oxidative stress, inflammation, and lipid metabolism as potential genetic markers for ALC [9, 10]. Notably, non-synonymous variants in the *Patatin-like phospholipase domain-containing protein 3*gene (*PNPLA3*; rs738409, I148M) and the *transmembrane 6 superfamily member 2* gene (*TM6SF2*; rs58542926, E167K) have been linked to the pathogenesis and progression of various liver diseases, irrespective of their etiology [11–14]. These proteins are crucial for lipid metabolism, and given that fatty liver is an initial manifestation of alcohol-related liver disease (ALD), it is hypothesized that they contribute to the development of cirrhosis [10].

The PNPLA3 protein functions as an enzyme with lipase activity toward triglycerides and retinyl esters, as well as acyltransferase activity on phospholipids. In humans, *PNPLA3* expression is most prominent in the liver, particularly in hepatocytes [15]. The I148M variant (rs738409) of *PNPLA3* is extensively studied due to its association with an increased risk of liver disease [16], linked to triglyceride accumulation in liver cells. This loss-of-function mutation involves a single nucleotide change from cytosine to guanine, resulting in an isoleucine-to-methionine substitution at position 148 (I148M) of the protein [17]. Carriers of this variant exhibit a heightened risk of developing steatosis and progressing to severe liver damage, including cirrhosis [18–20]. The TM6SF2 protein is involved in lipid export from liver cells, and lower levels of this protein are correlated with hepatic triglyceride accumulation and decreased secretion of very low-density lipoprotein triglycerides (VLDL TG) [21]. The *rs58542926* variant (E167K) in the *TM6SF2* gene, characterized by an adenine-to-guanine substitution at coding nucleotide 499, replaces glutamate at position 167 with lysine [14], and is associated with disrupted lipid metabolism, triglyceride accumulation, elevated serum aminotransferases, and decreased serum lipoproteins, contributing to fatty liver and potential progression to cirrhosis [22, 23].

Given the global rise in alcohol consumption, an increase in ALD cases is anticipated in the near future [24]. Preventing ALD involves the challenging task of reducing alcohol intake worldwide. Clinical experience indicates that informing patients of their elevated risk for the disease can lead to a reduction or cessation of alcohol consumption [25]. Thus, identifying excessive alcohol users at the highest risk for developing cirrhosis is critical for reducing the incidence of ALC.

### Aim of the study

This study aimed to genotype risk variants in the *PNPLA3* and *TM6SF2* genes in patients with ALC and evaluate their impact on ALC development by analyzing associated clinical and biochemical parameters, including standard liver function tests. We sought to investigate the relationship between *PNPLA3* and *TM6SF2* genetic variants, as well as a two-variant polygenic risk score (PRS), and the presence of ALC within our case-control cohort.

## Materials and methods

### Subjects

We recruited 118 patients diagnosed with ALC between 2015 and 2018 at the Clinic of Gastroenterology and Hepatology, University Hospital Medical Center “Zvezdara,” Belgrade, Serbia.

Cirrhosis was diagnosed based on a combination of clinical criteria, including laboratory parameters and clinical examination. Blood tests evaluating aspartate aminotransferase (AST), alanine aminotransferase (ALT), alkaline phosphatase (ALP), gamma-glutamyl transpeptidase (GGT), serum albumin, bilirubin levels, and coagulation parameters, including the international normalized ratio (INR), were conducted. Abnormal results from these tests may indicate liver failure.

Radiological confirmation of cirrhosis was obtained via ultrasonography and, if necessary, computed tomography, which identified nodular liver morphology, splenomegaly, collateral vessels, and ascites. Esophagogastroduodenoscopy assessed esophageal varices, while neuropsychological testing evaluated symptoms of hepatic encephalopathy, including confusion, asterixis, and fetor hepaticus. Each patient’s Child-Pugh score (CP) was calculated based on liver function test results and clinical examination findings to assess cirrhosis severity, classifying patients into A, B, and C classes, with Class C indicating advanced hepatic dysfunction and the poorest prognosis.

Standard serological and biochemical tests excluded other causes of liver disease (e.g., hepatitis C or B infections, autoimmune liver diseases, and metabolic liver diseases). Viral hepatitis was ruled out by screening for hepatitis B surface antigen (HBsAg), total anti-HBc, and anti-HCV antibodies. Autoimmune liver disorders were excluded based on negative findings for antinuclear (ANA), anti-smooth muscle (ASMA), and anti-mitochondrial (AMA) antibodies, along with normal serum immunoglobulin levels. Metabolic causes were excluded by measuring serum iron, ferritin, total iron-binding capacity, and transferrin saturation (for hereditary hemochromatosis); serum ceruloplasmin and 24-h urinary copper excretion (for Wilson’s disease); and serum α1-antitrypsin levels (for α1-antitrypsin deficiency). Patients with suspected drug-induced, cholestatic, or cryptogenic liver disease were excluded based on clinical history and biochemical findings.

Detailed information regarding age at onset of at-risk alcohol consumption, duration of at-risk alcohol consumption, daily alcohol intake, beverage type, and drinking patterns were collected during interviews at the initial clinical examination. The duration of at-risk alcohol consumption was calculated from self-reported age at the start of regular drinking to the age at cirrhosis diagnosis. An average daily consumption of more than 2 standard drinks for women and more than 3 standard drinks for men was classified as at-risk alcohol consumption. One standard drink is defined as a glass of beer, a glass of wine, or a shot of spirits, equating to 10 g of ethanol [26]. Average daily alcohol consumption in grams was determined by multiplying the volume of beverages by their alcohol strength and a conversion factor (0.789).

We selected 131 control subjects, matched for age and sex with cases, recruited from voluntary blood donors and individuals undergoing routine health examinations, who self-reported as either abstainers or individuals with daily alcohol consumption of less than one standard drink. Control subjects exhibited no signs of chronic liver disease and lacked a documented history of chronic hepatic illnesses or other primary pathological conditions. Laboratory assessments of standard liver function tests, including ALT, AST, ALP, and bilirubin, were within normal ranges, and no abdominal ultrasound or other imaging modalities were performed.

### Genotyping

Whole blood samples were collected from study participants in dipotassium ethylenediaminetetraacetic acid (K2EDTA)-coated vacutainers, and genomic DNA was extracted using a silica-membrane spin column-based isolation method, following the manufacturer’s protocol (Gene JET Whole Blood Genomic DNA Purification Mini Kit, Thermo Scientific, Massachusetts, USA). Isolated DNA was stored at –20 ^∘^C until further analysis. Genotyping for both investigated variants was performed using polymerase chain reaction followed by restriction fragment length polymorphism (PCR-RFLP) analysis. PCR reactions for each variant were conducted separately as previously described [27, 28]. Each PCR mixture (total volume 50 µL) contained 2xMultiplex Master Mix (Qiagen, Germany), 0.5 µM of each primer (Metabion, Germany), and 0.2 µg of genomic DNA. The PCR reactions were executed in a Tgradient thermal cycler (Biometra GmbH, Göttingen, Germany). The presence of PCR products was verified on a 2% agarose gel, stained with SYBR Safe DNA gel stain (Invitrogen, CA, USA), and visualized under UV light. Restriction endonucleases BseGI (BtsCI) for *PNPLA3* (rs738409 C>G) and Hpy188I for *TM6SF2* (rs58542926 C>T) (New England BioLabs, MA, USA) were used to digest the PCR products according to the manufacturer’s instructions. The digested fragments were then electrophoresed on a 3% agarose gel to determine genotypes based on fragment length (see Table S1).

All subjects were successfully genotyped for both tested variants. To validate the quality of the procedure, 10% of samples were randomly selected for re-genotyping using the same PCR-RFLP methods. Samples were processed in multiple laboratory batches, ensuring a balanced distribution of cases and controls across batches. Throughout all phases of DNA processing (PCR, digestion, and gel scoring), laboratory personnel were blinded to case–control status. Any discrepancies, including sex mismatches and potential sample swaps, were resolved by two independent investigators (one clinician and one scientist) through verification of consistency between participant records and laboratory documentation.

### Ethical statement

All patients and control subjects reported Serbian ancestry. All procedures were conducted in accordance with the Helsinki Declaration of 1975, and the study protocol was approved by the Ethics Committee of the University Hospital Medical Center “Zvezdara” (Approval No. 8-6-2018, dated June 1, 2018). Informed consent was obtained from all study participants.

### Statistical analysis

Data analysis was performed using the Statistical Package for the Social Sciences (SPSS version 20.0, SPSS Inc., Chicago, USA). A *P* value of <0.05 was considered statistically significant. To address multiplicity, the Benjamini–Hochberg (BH) false discovery rate (FDR) method was applied, with statistical significance defined at FDR ≤ 0.05 and secondary significance at FDR ≤ 0.1. Allele and genotype frequencies were determined through direct counting. Hardy–Weinberg equilibrium (HWE) was assessed for both ALC patients and the control group. Continuous variables are presented as means with standard deviations or medians with 25th and 75th percentiles, depending on their distribution normality, which was tested using both mathematical and graphical methods. Group differences for normally distributed continuous variables were analyzed using independent *t*-tests and one-way ANOVA as appropriate. The Kruskal–Wallis test was employed for non-normally distributed variables. Categorical variables are presented as counts and percentages. The chi-square or Fisher’s exact test, as appropriate, was used to evaluate differences between groups for categorical variables. Effect sizes and 95% confidence intervals (CIs) were generated using a nonparametric bootstrap procedure with 2000 resamples, performed in Python (SciPy, pandas). Correlations between variables were analyzed using Pearson’s and point-biserial correlations as applicable.

The PRS based on the two selected variants was computed using software R v.4.3.0 with the following function:

f_β(x) ═ (Σ_{i=1}ˆ{n}β_i · g(x_i))/(Σ_{i=1}ˆ{n}β_i)

For each gene, g(xi) represented points of 0, 0.5, and 1 assigned to participants based on their genotype. Individuals with two reference alleles received 0 points, heterozygous carriers of the risk allele associated with ALC received 0.5 points, and homozygous carriers of the risk allele received 1 point. The maximum g(xi) value was 2 if an individual was homozygous for both assessed variants. Furthermore, β coefficients representing allelic effect weights were obtained from the PGS Catalog searched for the term alcoholic liver cirrhosis (PRS ID: PGS000704; https://www.pgscatalog.org/score/PGS000704; accessed on April 8, 2025) [29]. Effect weights for the tested variants are detailed in Table 1. Differences in PRS distribution between controls and ALC patients were assessed using a non-parametric Wilcoxon rank sum test for continuous data. The association between genotypes/PRS and ALC was evaluated using binary logistic regression. Results are expressed as odds ratios (ORs) with 95% CIs. Due to exclusion criteria for control subjects regarding diabetes and alcohol use, as well as the influence of cirrhosis on body mass index (BMI), these variables were not included as confounding factors to prevent bias. The association between *TM6SF2* genotypes and ALC was assessed using Firth regression to address sparse genotype categories (few *TM6SF2* TT genotypes). To comprehensively evaluate potential inheritance patterns, each variant was analyzed using additive, dominant, and recessive models. Participants were stratified into three balanced PRS-based groups (tertiles) according to empirical distribution of PRS values for the assessment of the association of PRS with ALC. The reference group (low-risk group) consisted of patients with PRS=0, including subjects with wild-type (wt) genotypes for both tested variants. Receiver operating characteristic (ROC) curve analysis was used to evaluate the performance of the PRS model.

**Table 1:** Parameters utilized in the polygenic risk score analysis

Genetic variant (risk allele)	***PNPLA3* rs738409 (G)**	***TM6SF2* rs58542926 (T)**
β (logOR)	0.19895	0.186567

A risk allele refers to the genetic variant associated with alcohol-related liver cirrhosis. In this study, homozygous carriers of alleles linked to alcohol-related liver cirrhosis received 1 point for each gene analyzed, while heterozygous carriers were assigned 0.5 points, and non-carriers received 0 points. The β values utilized in this research were obtained from the PGS Catalog (PRS ID: PGS000704, accessed on April 8, 2025). Abbreviations: PNPLA3: Patatin-like phospholipase domain-containing protein 3; TM6SF2: Transmembrane 6 superfamily member 2; β: Regression coefficient; OR: Odds ratio; logOR: Natural logarithm of the odds ratio; PRS: Polygenic risk score; PGS: Polygenic score.

**Figure 1.**
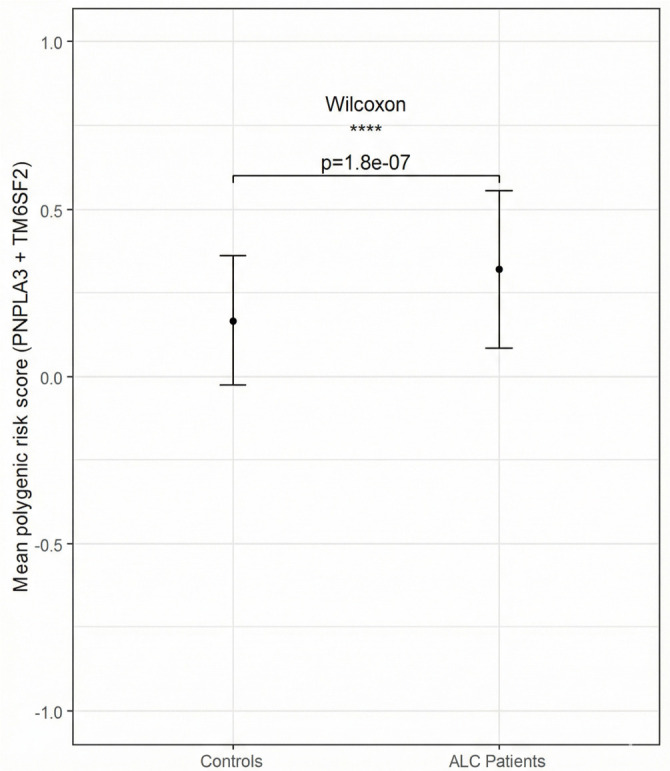
**Distribution of PRS in ALC patients and control subjects.** The means for the control and ALC groups are denoted by dots (0.17 for the control group, 0.32 for the ALC group). Error bars indicate the standard deviation (0.19 for the control group, 0.23 for the ALC group). Given that the standard deviation exceeded the mean in the control group, suggesting a non-normal distribution, differences were analyzed using the non-parametric Wilcoxon rank sum test for continuous data. Abbreviations: PRS: Polygenic risk score; ALC: Alcohol-related liver cirrhosis.

Internal validation was conducted using 1000 bootstrap resamples, yielding optimism-corrected area under the curve (AUC) and 95% CI. Model calibration was examined using the calibration slope and the Hosmer-Lemeshow test. The same β-weighted and normalized PRS was utilized consistently across all analyses, including Wilcoxon comparisons, Figure 1, tertile classification, and ROC analyses. For bootstrap validation and calibration slope estimation, Python 3.13 with pandas, NumPY, and scikit-learn libraries was employed. All analyses were restricted to complete cases.

## Results

Our study involved 249 participants, including 118 patients diagnosed with ALC and 131 control subjects. Gender representation was comparable in both groups (*P* ═ 0.540); the ALC group consisted of 104 men (88.1%) and 14 women (11.9%), while the control group included 112 men (85.5%) and 19 women (14.5%). The mean age of ALC patients was 58.6 ± 9.6 years (ranging from 32 to 80 years), whereas the mean age in the control group was 58.4 ± 10.8 years (ranging from 30 to 87), with no statistically significant difference observed (*P* ═ 0.846). The median age at the onset of at-risk alcohol consumption among ALC patients was 23 years, with a median daily alcohol intake of 72 g and an average duration of alcohol consumption prior to cirrhosis diagnosis of 34.6 ± 10.98 years. Additional characteristics of the ALC patients are summarized in Table 2.

**Table 2:** Characteristics of ALC patients

**Drinking profile**			**Clinical characteristics**			**Laboratory parameters**	
Age at onset of at-risk alcohol consumption, years		23 (19–30)	Diabetes, N (%)		32 (27.1)	AST, IU/L	68.5 (43.5–113)
Duration of at-risk alcohol consumption, years		34.54 (10.98)	Child-Pugh class	A, N (%)	18 (15.3)	ALT, IU/L	36 (22–54.25)
Daily alcohol consumption, g		72 (56–90)		B, N (%)	47 (39.8)	ALP, IU/L	117 (79.75–156.5)
Type of beverage	Beer, N (%)	82 (69.5)		C, N (%)	53 (44.9)	GGT, IU/L	128 (70–261.25)
	Wine, N (%)	25 (21.2)	Ascites, N (%)		84 (71.2)	Albumin, g/L	28 (25–32.25)
	Spirits, N (%)	87 (73.7)	Encephalopathy, N (%)		63 (53.4)	Bilirubin, µmol/L	43.7 (21.35–90.8)
			Esophageal varices, N (%)		71 (60.2)	INR	1.5 (1.30–1.79)

The data are presented as means ± standard deviations or medians (25th - 75th percentiles), unless otherwise specified. It is important to note that beverage categories are non-mutually exclusive, resulting in percentages that may exceed 100%. Abbreviations: N: Number of subjects; AST: Aspartate aminotransferase; ALT: Alanine aminotransferase; ALP: Alkaline phosphatase; GGT: Gamma-glutamyl transpeptidase; INR: International normalized ratio.

Pearson’s correlation coefficient was utilized to assess the relationship between variables associated with alcohol consumption. Patients who initiated at-risk alcohol consumption at an older age demonstrated a more rapid progression to cirrhosis (r(118) ═ –0.513; *P* < 0.001). However, no significant correlation was found between the median daily alcohol consumption (72 g/day) and either the duration of alcohol consumption prior to ALC diagnosis or the age at which at-risk alcohol consumption began. A point-biserial correlation analysis explored the relationship between average daily alcohol consumption and beverage type. Results indicated that patients consuming spirits, either exclusively or in combination with other beverages, had a higher daily alcohol intake (rpb = 0.262, *n* ═ 116, *P* ═ 0.004).

### Association of variant genotypes *PNPLA3* rs738409 and *TM6SF2* rs58542926 with ALC development

The frequency of the G allele of the *PNPLA3rs738409* variant was 0.45 in the ALC group and 0.24 in the control group. The prevalence of the T allele of the *TM6SF2rs58542926* variant was significantly higher in patients (0.177) compared to control subjects (0.084) (*P* ═ 0.002). The GG genotype of *PNPLA3* was more frequently observed in ALC patients than in the control group (*P* < 0.001). Conversely, the CC genotype of the *TM6SF2* variant was more prevalent in the control group (84.7%) than in the ALC group (68.7%, *P* ═ 0.003). The CT genotype was more common in the ALC group compared to the control group (27.1% vs 13.8%, *P* ═ 0.009). The genotype distributions for each studied variant adhered to HWE (Table S2). The G allele in *PNPLA3* and the T allele in *TM6SF2* were strongly associated with ALC (*P* < 0.001 and *P* ═ 0.002, respectively, as shown in Table 3). We further examined the relationship between genotypes of the studied genes and ALC, incorporating sex and age as covariates in multiple logistic regression models. Subjects with CG and GG genotypes of *PNPLA3* exhibited an almost twofold and nearly eightfold increased risk of developing cirrhosis, respectively, compared to the CC genotype (CG: OR=1.82; 95% CI=1.05–3.17; *P* ═ 0.033; GG: OR=7.64; 95% CI=3.06–19.07; *P* < 0.001). As detailed in Table 3, the *PNPLA3* variant was significantly associated with ALC under both dominant and recessive models (*P* < 0.001 for both). Given the limited occurrence of the TT genotype in the *TM6SF2* gene (five patients and two controls; *P* ═ 0.261), we employed Firth logistic regression. Subjects with the CT genotype of *TM6SF2* had a 2.43-fold increased risk for ALC (OR=2.43; 95% CI=1.27–4.63; *P* ═ 0.007). The dominant genetic model indicated that carriers of the CT or TT genotype of *TM6SF2* had a 2.5-fold higher likelihood of developing cirrhosis compared to the CC genotype (OR=2.52; 95% CI=1.36–4.66; *P* ═ 0.003). The findings are summarized in Table 3. After adjusting for multiple testing using the BH method at FDR=0.05, the BH thresholds were *P* ≤ 0.0375 for the χ^2^ tests and *P* ≤ 0.0444 for the regression models.

**Table 3:** Association of analyzed alleles/genotypes in variants *PNPLA3* rs738409 and *TM6SF2* rs58542926 with the development of alcohol-related liver cirrhosis

	**Odds ratio [95% CI]**	***P* value**
*PNPLA3*		
G vs C	2.55 [1.74–3.74]	<0.001
CG vs CC	1.82 [1.05–3.17]	0.033
GG vs CC	7.64 [3.06–19.07]	<0.001
Dominant model CG/GG vs CC	2.54 [1.51–4.26]	<0.001
Recessive model GG vs CC/CG	5.73 [2.4–13.7]	<0.001
*TM6SF2*		
T vs C	2.34 [1.35–4.06]	0.002
CT vs CC	2.43 [1.27–4.63]	0.007^*^
TT vs CC	3.33 [0.63–17.68]	0.158^*^
Dominant model CT/TT vs CC	2.52 [1.36–4.66]	0.003
Recessive model TT vs CC/CT	2.76 [0.52–14.58]	0.232^*^

The odds ratio is adjusted for sex and age. The 95% CI and *P* value are reported. Although only raw *P* values are presented, multiple testing was controlled using the Benjamini–Hochberg procedure, with a FDR set at 0.05. The significance threshold for the Benjamini–Hochberg procedure was *P* ≤ 0.0444. *P values are derived from Firth logistic regression. Abbreviations: PNPLA3: Patatin-like phospholipase domain-containing protein 3; TM6SF2: Transmembrane 6 superfamily member 2; CI: Confidence interval; FDR: False discovery rate.

### Estimation of ALC development risk using a PRS

We generated variant-based PRS for the two tested variants, assessing the risk of developing ALC by comparing scores between the studied groups. The distribution of PRS among ALC patients and control subjects was statistically significant (*P* ═ 1.8e-07). The mean PRS for ALC patients was 0.32, while the control group had a mean PRS of 0.167 (Figure 1). For logistic regression analysis, patients were categorized into three risk groups (low, moderate, high) based on their PRS values. The reference group (low risk) consisted of patients with a PRS of 0, comprising individuals with wild-type genotypes for both tested variants. The high-risk group exhibited a sevenfold increased likelihood of developing ALC compared to the reference group after adjusting for age and sex (*P* < 0.001) (Table 4). The model demonstrated moderate discriminatory power, with an AUC of 0.684 (95% CI 0.617–0.750). Internal validation using 1000 bootstrap resamples yielded an optimism-corrected AUC of 0.684 (95% CI: 0.616–0.745), indicating minimal overfitting and high stability of model performance. The calibration slope of 1.360 suggested mild underfitting, and the Hosmer-Lemeshow goodness-of-fit test indicated excellent concordance between observed and predicted risk (χ^2^ ═ 3.441; *P* ═ 0.904).

**Table 4:** Adjusted ORs for the two-SNP polygenic risk score (*PNPLA3***+***TM6SF2***)**

**Risk group**	**Score**	Odds ratio^*^	**Lower 95% CI**	**Upper 95% CI**	***P* value**
Low, *N* ═ 94	0	Reference			
Moderate, *N* ═ 87	>0 ≤ 0.26	1.731	0.937	3.199	0.080
High, *N* ═ 68	>0.26	6.707	3.313	13.581	<0.001

*The odds ratio is adjusted for sex and age. A threshold of 0.26 represents the first tertile cut-off from the empirical PRS distribution. The low-risk group comprises subjects with a PRS of 0 or carriers of wild-type (wt) genotypes for both tested variants. The moderate-risk group includes subjects with a PRS greater than 0 but less than 0.26. The high-risk group consists of subjects with a PRS greater than 0.26. Abbreviations: OR: Odds ratio; SNP: Single-nucleotide polymorphism; PNPLA3: Patatin-like phospholipase domain-containing protein 3; TM6SF2: Transmembrane 6 superfamily member 2; N: Number of subjects; CI: Confidence interval; PRS: Polygenic risk score.

### Clinical characteristics of the ALC group according to *PNPLA3* and *TM6SF2* genotypes

This study compared drinking profiles, laboratory parameters, and clinical characteristics of ALC patients based on their *PNPLA3* rs738409 and *TM6SF2* rs58542926 genotypes. The average daily alcohol consumption significantly differed among the *PNPLA3* genotypes, with CC genotype carriers consuming the highest amounts compared to CG and GG genotype carriers (*P* ═ 0.002). A notable trend indicated that higher levels of ALT were associated with an increasing number of *G* alleles in the *PNPLA3* gene (Kruskal–Wallis H(2) ═ 8.10, *P* ═ 0.017). The effect size was η^2^ ═ 0.052, with a bootstrap CI ranging from --0.005 to 0.182, suggesting a small to moderate effect. However, this association lost statistical significance upon applying the BH procedure at both FDR = 0.05 and FDR = 0.1. Other laboratory parameters did not differ significantly between subgroups (*P* > 0.05 for all parameters, Table 5). Similarly, no significant differences were observed in clinical characteristics across the *TM6SF2* genotypes (*P* > 0.05 for all parameters, Table 5).

**Table 5:** Comparison of characteristics of ALC patients across *PNPLA3* and *TM6SF2* genotypes

		***PNPLA3***			***P* value**	***TM6SF2***			***P* value**
		**CC (N ═ 40)**	**CG (N ═ 49)**	**GG (N ═ 29)**		**CC (N ═ 81)**	**CT (N ═ 32)**	**TT (N ═ 5)**	
Gender, *n* (%)					0.067				0.315
Female		7 (17.5)	7 (14.3)	0 (0)	12 (14.8)	2 (6.3)	0 (0)		
Male		33 (82.5)	42 (85.7)	29 (100)		69 (85.2)	30 (93.8)	5 (100)	
Age		60.25 (8.96)	58.0 (9.3)	57.5 (10.8)	0.424	58.5 (9.6)	58.1 (9.9)	63.3 (6.6)	0.522
*Drinking profile*									
Age at onset of at-risk alcohol consumption, years		23.5 (19.2–30)	23 (19–28)	21 (18–25)	0.446	22 (19–27.5)	24 (18.2–30)	25 (19–30.5)	0.824
Duration of at-risk alcohol consumption, years		35.1 (11.2)	33.7 (11)	35.0 (11)	0.774	34.6 (10.6)	33.8 (12.4)	38.5 (8.9)	0.674
Daily alcohol consumption, g		80 (60–100)	60 (37–75)	74 (48–100)	**0.002**	72 (48–90)	75 (58.7–100)	60 (45–80)	0.611
Type of beverage	Beer, *n* (%)	29 (72.5)	33 (67.3)	20 (69)	0.766	57 (70.4)	21 (65.6)	4 (80)	0.745
	Wine, *n* (%)	9 (22.5)	10 (20.4)	6 (20.7)	0.969	21 (25.9)	4 (12.5)	0 (0)	0.144
	Spirits, *n* (%)	32 (80)	34 (69.4)	21 (72.4)	0.518	61 (75.3)	22 (68.8)	4 (80)	0.735
*Laboratory parameters*									
AST, IU/L		63 (36.8–119)	59 (41–100)	84 (51.5–127)	0.277	66 (42–111.5)	68.5 (44.2–112.5)	72 (39–377)	0.876
ALT, IU/L		30.5 (21.3–41.5)	35 (22.5–57.5)	47 (32–72)	**0.017**	36 (22–50)	36 (22.2–57.2)	30 (19.5–421)	0.863
ALP, IU/L		120.5 (78.3–157)	110 (79.5–154.5)	115 (76–171.5)	0.958	122 (81.5–159.5)	106 (74–154.7)	104 (79.5–269)	0.657
GGT, IU/L		141 (71–226)	120 (54.5–301.5)	131 (84–392)	0.555	124 (70.5–249)	136.5 (71–274.7)	131 (38–400)	0.912
Albumin, g/L		29 (26.0–32.8)	27 (25–32.5)	27 (24–32)	0.465	28 (25–32)	27 (24–34.7)	28 (26.5–32.5)	0.951
Bilirubin, µmol/L		42.65 (22.4–88.4)	43.8 (20.2–111.4)	40.8 (21.3–90.5)	0.907	40.8 (22.2–90)	50.2 (19.2–91.5)	58.5 (23.3–196)	0.835
INR		1.48 (1.3–1.8)	1.52 (1.3–1.7)	1.42 (1.3–1.8)	0.897	1.44 (1.3–1.7)	1.51 (1.2-1.8)	1.7 (1.3-2.3)	0.636
*Clinical characteristics*									
Diabetes N (%)		9 (22.5)	12 (24.5)	11 (37.9)	0.314	21 (25.9)	10 (31.3)	1 (20)	0.793
Child-Pugh class	A, N (%)	6 (15)	7 (14.3)	5 (17.2)	0.939	11 (13.6)	6 (18.8)	1 (20)	0.754
	B, N (%)	15 (37.5)	20 (40.8)	12 (41.4)	0.933	37 (45.7)	9 (28.1)	1 (20)	0.149
	C, N (%)	19 (47.5)	22 (44.9)	12 (41.4)	0.88	33 (40.7)	17 (53.1)	3 (60)	0.386
Ascites, N (%)		27 (67.5)	37 (75.5)	20 (69)	0.677	61 (75.3)	19 (59.4)	4 (80)	0.219
Encephalopathy, N (%)		26 (65)	23 (46.9)	14 (48.3)	0.193	41 (50.6)	18 (56.3)	4 (80)	0.411
Esophageal varices, N (%)		22 (55)	35 (71.4)	14 (48.3)	0.093	49 (60.5)	19 (59.4)	3 (60)	0.994

The data are presented as means ± standard deviations or medians (25th–75th percentile), unless otherwise specified. Beverage categories are non-mutually exclusive; therefore, percentages may exceed 100%. Normally distributed continuous variables were evaluated using the independent *t*-test and one-way ANOVA, as appropriate. For non-normally distributed variables, the Kruskal–Wallis Test was employed. The chi-square test or Fisher’s exact test, as appropriate, was utilized to assess differences between groups for categorical variables. Multiple testing was controlled using the Benjamini–Hochberg procedure. None of the *P* values met the significance criteria at FDR of 0.05 or 0.1. Abbreviations: PNPLA3: Patatin-like phospholipase domain-containing protein 3; TM6SF2: Transmembrane 6 superfamily member 2; P: Probability; N: Number of subjects; AST: Aspartate aminotransferase; ALT: Alanine aminotransferase; ALP: Alkaline phosphatase; GGT: Gamma-glutamyl transpeptidase; INR: International normalized ratio; FDR: False discovery rates.

## Discussion

ALC is a multifactorial disease characterized by considerable variability in progression and outcomes, influenced by both environmental and genetic factors and their interactions. Alcohol intake [30, 31] and drinking patterns [4, 5] have been shown to correlate directly with the risk of liver disease. Women exhibit comparable effects to men at lower levels of alcohol consumption [6, 32]. Furthermore, genetic risk factors, along with chronic and acute conditions, may influence the progression of ALD [2, 33].

The *PNPLA3* rs738409 variant has been consistently associated with various liver pathologies, including steatosis, fibrosis, cirrhosis, and hepatocellular carcinoma (HCC), across different etiologies [12, 16, 17, 20, 34–37]. This variant promotes lipid accumulation in the liver by enhancing the conversion of lysophosphatidic acid into phosphatidic acid, resulting in increased lipotoxicity [38]. An initial association between this variant and hepatic fat concentration was identified in studies on metabolic dysfunction-associated steatotic liver disease (MASLD) [17]. Subsequent research established a strong correlation between this variant and ALD [20], ALC [12], and HCC [34]. Our findings indicate that carriers of both the CG and GG genotypes of the *PNPLA3* rs738409 variant are at a higher risk of developing cirrhosis compared to CC genotype carriers, thereby reinforcing the association of this variant with ALC, consistent with previous studies [18, 20, 39–42]. The *TM6SF2* gene has also emerged as a candidate gene for various liver diseases [12]. Protein TM6SF2 plays a role in very-low-density lipoprotein (VLDL) secretion. The rs58542926 variant has been linked to increased intracellular lipid accumulation, contributing to fatty liver, ALC [12], HCC [13], and MASLD [43]. Our results demonstrated that the likelihood of developing cirrhosis was more than twofold higher in carriers of the CT or TT genotype (compared to CC) of the *TM6SF2* variant rs58542926. Notably, we identified only seven individuals with the TT genotype (5 ALC patients and 2 control subjects). These findings align with previous reports [13]. The frequencies of the G allele of the *PNPLA3* rs738409 variant and the T allele of the *TM6SF2* variant rs58542926 were elevated in cirrhotic patients, indicating that carriers of these alleles are at an increased risk for the disease. The allele frequencies of *PNPLA3* and *TM6SF2* in our control group correspond to those reported for the European population in the Genome Aggregation Database (gnomAD) [44, 45]. However, the interpretation of the effects of *TM6SF2* is limited by the low number of TT homozygotes, which resulted in wide CIs and unstable estimates in the analysis. Thus, these findings should be regarded as exploratory, and validation of the TT-associated risk necessitates larger or pooled cohorts.

We further assessed the cumulative impact of the examined variants through PRS calculations to identify individuals at risk for developing ALC. Our study revealed that the mean PRS was twofold higher in the ALC patient group compared to the control group. Stratification of subjects into three risk categories based on PRS values allowed for more precise identification of individuals at the highest risk of developing cirrhosis; those in the highest score group had a sevenfold increased risk of ALC relative to the reference group. Although the AUC value of 0.684 indicates moderate predictive accuracy, this level of discrimination is consistent with findings from other studies [46]. The PRS model demonstrated stable performance and good calibration within the internal dataset. A broader panel of variants for PRS calculations, including comorbidities associated with ALC, such as diabetes mellitus, has been reported [46–48]. Whitfield et al. [46] demonstrated that a three-variant risk score, when combined with diabetes, improved discrimination of ALC among heavy alcohol consumers. Additionally, a 20-single-nucleotide polymorphism (SNP) PRS further enhanced prediction when integrated with clinically known predictors of ALC risk in the drinking population [48]. In contrast, our study utilized only two nucleotide variants, excluded diabetes and BMI, and incorporated non-drinking controls, thereby estimating genetic susceptibility without the confounding effects of alcohol exposure or metabolic comorbidities. This model facilitates the identification of genetic associations and simple risk stratification within our case-control study. While it suggests potential for earlier risk identification in broader populations, the application of these genetic variants and our simple PRS for early stratification among heavy drinkers or prediction prior to heavy drinking initiation remains hypothetical and warrants further investigation. Although simpler and potentially less discriminative in high-risk alcohol drinkers, the 2-SNP model presents a practical and cost-effective approach that could be integrated into broader multivariable risk-prediction tools.

We analyzed the relationship between genotypes and characteristics of patients with ALC. Carriers of the CC genotype of the *PNPLA3* gene variant rs738409 exhibited a lower risk for ALC, despite significantly higher daily alcohol consumption, compared to carriers of other genotypes. Conversely, individuals carrying one or two G alleles were more susceptible to cirrhosis, even with lower alcohol intake. This finding aligns with previous research indicating that individuals with this variant are at an increased risk of developing fatty liver and cirrhosis [18–20].

As the number of G alleles of the *PNPLA3* rs738409 increased, a significant trend toward elevated serum levels of ALT was observed. However, this association did not remain statistically significant after applying the BH correction for multiple comparisons. The effect size was small to moderate, necessitating cautious interpretation of these results. Elevated ALT levels have previously been associated with *PNPLA3* genotypes [49, 50]. ALT serves as a highly sensitive and specific marker of liver function, localized in hepatocytes, yet the precise mechanism by which *PNPLA3* influences ALT elevation remains unclear [51]. One possible explanation is that a dysfunctional protein may lead to fat accumulation in the liver, resulting in increased liver enzyme levels or heightened ALT due to hepatocyte necrosis and impaired liver function.

No significant differences in other biochemical parameters were observed across the genotypes, suggesting that the biochemical markers examined were not associated with the genetic variants explored in our study.

It is important to acknowledge that this research is a single-center study; future investigations should aim for larger sample sizes to enhance the generalizability of the findings. Our patients were diagnosed without liver biopsy. Although liver biopsy is considered the gold standard for diagnosing cirrhosis, many patients with chronic liver disease can be accurately diagnosed using non-invasive methods [52]. In our study, diagnoses were based on clinical symptoms, laboratory test results, and a history of long-term excessive alcohol consumption, while excluding other potential causes of cirrhosis.

The control group was restricted to individuals without diabetes or significant alcohol consumption to ensure a homogeneous comparison. Since alcohol intake and diabetes were exclusion criteria for one group, and BMI was influenced by cirrhosis, incorporating these variables into regression models would have introduced bias. Consequently, only sex and age adjustments were used in the regression models. A limitation of the study design is the inability to exclude gene-environment interactions. The estimated ORs for variants and PRS should be interpreted as associations within this study framework, rather than fully independent genetic effects. Recruiting individuals with significant alcohol consumption as controls would allow for demonstrating an independent association of variants with cirrhosis, adjusted for alcohol intake. However, this approach would necessitate liver biopsy for control participants to prevent misclassification, which was not feasible. While these design choices limit generalizability, they were essential for a clear assessment of genotype associations within a defined population. Another concern regarding the study participants is the potential inaccuracy of self-reported alcohol consumption data, which may be influenced by recall bias or intentional misreporting. Alcohol intake is often underreported due to consumer awareness of its harmful effects and social desirability [53]. It is reasonable to assume that patients with cirrhosis who report ongoing drinking are providing accurate information, whereas those reporting abstinence or low alcohol consumption may include individuals consuming excessive amounts. Our PRS represents an initial proof-of-concept model that should be further optimized for clinical use. To effectively identify at-risk patients, the risk score should incorporate additional genetic variants alongside other known risk factors.

## Conclusion

Understanding the genetic underpinnings of chronic liver diseases and identifying an individual’s genetic susceptibility is crucial for developing management strategies, including lifestyle modifications and monitoring for disease progression. In our cohort, *PNPLA3* rs738409 and *TM6SF2* rs58542926 variants were associated with an elevated risk of ALC, and a PRS based on these loci effectively identified individuals with increased genetic susceptibility to the disease. Given the case-control design, these findings represent preliminary cohort-specific risk associations. Future research involving heavy-drinking controls without ALC will be necessary to assess disease severity and the potential clinical significance of this two-variant PRS.

## Supplemental data

**Table S1:** PCR-RFLP conditions and restriction fragment patterns for analyzed *PNPLA3* and *TM6SF2* variants

**Gene (variant)**	**Forward primer (5′→3′)** **Reverse primer (5′→3′)**	**PCR conditions (35 cycles)**	**PCR product size (bp)**	**Restriction enzyme**	**Fragment pattern (bp)**
*PNPLA3* (rs738409)	TGGGCCTGAAGTCCGAGGGT CCGACACCAGTGCCCTGCAG	ID:94^∘^C/2 min; D:94^∘^C/30 s; A:66^∘^C/30 s; E:72^∘^C/30 s; FE:72^∘^C/5 min	333	BseGI (BtsCI)	C allele: 200+133 G allele: 333
*TM6SF2* (rs58542926)	ACGGGGAAAGTTCAGGCACATTG CCTGGGCAGCATGGTGAAACC	ID:94^∘^C/2 min; D:94^∘^C/30 s; A:62^∘^C/30 s; E:72^∘^C/30 s; FE:72^∘^C/5 min	429	Hpy188I	C allele: 178+33+85 T allele: 251+178

Abbreviations: ID: Initial denaturation; D: Denaturation; A: Annealing; E: Elongation; FE: Final elongation; PCR-RFLP: Polymerase chain reaction–restriction fragment length polymorphism; bp: Base pairs; PNPLA3: Patatin-like phospholipase domain-containing protein 3; TM6SF2: Transmembrane 6 superfamily member 2.

**Table S2:** Genotype distribution and allele frequencies of variants *PNPLA3* rs738409 and *TM6SF2* rs58542926 in the ALC and control group

**Variant**	**Allele/genotype**	**ALC group**		**Control group**		***P***
		***N* ═ 118**		***N* ═ 131**		
*PNPLA3*	C, N (%)	129	(54.7)	198	(75.6)	**<0.001**
	G, N (%)	107	(45.3)	64	(24.4)	
	CC, N (%)	40	(33.9)	74	(56.5)	**<0.001**
	CG, N (%)	49	(41.5)	50	(38.2)	0.589
	GG, N (%)	29	(24.6)	7	(5.3)	**<0.001**
HWE^p^		0.078		0.699		
*TM6SF2*	C, N (%)	194	(82.2)	240	(91.6)	**0.002**
	T, N (%)	42	(17.8)	22	(8.4)	
	CC, N (%)	81	(68.7)	111	(84.7)	**0.003**
	CT, N (%)	32	(27.1)	18	(13.8)	**0.009**
	TT, N (%)	5	(4.2)	2	(1.5)	0.261
HWE^p^		0.427		0.221		

Differences between allele and genotype frequencies were assessed using the chi-square test or Fisher’s exact test in cases of sparse cell counts. Raw *P* values are presented. Multiple testing corrections were applied using the Benjamini–Hochberg procedure (FDR = 0.05), with results deemed significant at *P* values ≤ 0.0375. Abbreviations: ALC: Alcohol-related liver cirrhosis; N: Number of subjects; HWE^p^: Hardy–Weinberg equilibrium *P* value; FDR: False discovery rates.

## Data Availability

The data are available only upon request, due to a non-disclosure agreement provided by the institution.
